# A resource-poor developmental diet reduces adult aggression in male *Drosophila melanogaster*

**DOI:** 10.1007/s00265-021-03050-z

**Published:** 2021-07-22

**Authors:** Danielle Edmunds, Stuart Wigby, Jennifer C. Perry

**Affiliations:** 1 Department of Zoology, University of Oxford, Oxford, UK; 2 Department of Evolution, Ecology, and Behaviour, Institute of Infection, University of Liverpool, Veterinary & Ecological Sciences, Liverpool, UK; 3 School of Biological Sciences, University of East Anglia, Norwich, NR4 7TJ UK

**Keywords:** Aggression, Development, Diet, *Drosophila melanogaster*, Nutrition

## Abstract

Aggressive behaviours occur throughout the animal kingdom and agonistic contests often govern access to resources. Nutrition experienced during development has the potential to influence aggressive behaviours in adults through effects on growth, energy budgets and an individual’s internal state. In particular, resource-poor developmental nutrition might decrease adult aggression by limiting growth and energy budgets, or alternatively might increase adult aggression by enhancing motivation to compete for resources. However, the direction of this relationship—and effects of developmental nutrition experienced by rivals—remains unknown in most species, limiting understanding of how early-life environments contribute to variation in aggression. We investigated these alternative hypotheses by assessing male-male aggression in adult fruit flies, *Drosophila melanogaster*, that developed on a low-, medium- or high-resource diet, manipulated via yeast content. We found that a low-resource developmental diet reduced the probability of aggressive lunges in adults, as well as threat displays against rivals that developed on a low-resource diet. These effects appeared to be independent of diet-related differences in body mass. Males performed relatively more aggression on a central food patch when facing rivals of a low-resource diet, suggesting that developmental diet affects aggressive interactions through social effects in addition to individual effects. Our finding that resource-poor developmental diets reduce male-male aggression in *D. melanogaster* is consistent with the idea that resource budgets mediate aggression and in a mass-independent manner. Our study improves understanding of the links between nutrition and aggression.

**Significance statement**

Early-life nutrition can influence social behaviours in adults. Aggression is a widespread social behaviour with important consequences for fitness. Using the fruit fly, *Drosophila melanogaster*, we show that a poor developmental diet reduces aspects of adult aggressive behaviour in males. Furthermore, males perform more aggression near food patches when facing rivals of poor nutrition. This suggests that early-life nutrition affects aggressive interactions through social effects in addition to individual effects.

**Supplementary Information:**

The online version contains supplementary material available at 10.1007/s00265-021-03050-z.

## Introduction

Aggression is widespread amongst animals (e.g. mammals, Sinn et al. 2008; birds, Johnsen and Zuk 1995; fish, Neat et al. 1998; Seebacher et al. 2013; invertebrates, Brown et al. 2007; Elias et al. 2010), including humans, where aggressive behaviours have detrimental effects on societies (Blanchard and Blanchard 2003; Sluyter et al. 2003; Georgiev et al. 2013). Success in aggressive contests can provide superior access to critical reproductive resources such as food, territories and mates (Clutton-Brock and Albon 1979; Hoffman 1987; Huntingford et al. 2012; Georgiev et al. 2013; Belenioti and Chaniotakis 2020), but contests come with costs, including physical damage, time and energy expenditure and increased predation risk (Haley 1994; Neat et al. 1998; Briffa and Sneddon 2007). Individuals often display temporary, reversible changes in aggressive behaviours throughout life (Huntingford et al. 2012; Georgiev et al. 2013), and much of this variation is likely to result from differences in an individual’s environment (Dochtermann et al. 2015; Han and Dingemanse 2017; Bath et al. 2021). Understanding the ecological factors that determine aggressive behaviour can help to elucidate its evolution and consequences.

Nutrient availability and quality are key components of an individual’s environment and can shape behavioural strategies expressed throughout life (Lihoreau et al. 2015). In many species, early life is key for nutrient acquisition, and the balance of nutrients in this critical period can have profound effects on body mass, resource allocation to adult traits, and internal state (Royle et al. 2005; Amitin and Pitnick 2007; Zikovitz and Agrawal 2013; Lihoreau et al. 2015; Pillay et al. 2016; Han and Dingemanse 2017). These effects can determine the relative ability (i.e. resource-holding potential) and motivation (i.e. resource valuation, the value of a contested resource; Elias et al. 2010; Stockermans and Hardy 2013; Gruber et al. 2016) to invest in aggressive contests and the fitness pay-offs from doing so. Thus, individuals should benefit from moderating aggression adaptively in response to nutritionally derived cues experienced in early life (Scharf 2016). Consistent with this prediction, there is evidence that early-life diet influences levels of aggression and antisocial behaviours in humans and non-human vertebrates (Wallner and Machatschke 2009).

However, the net effect of developmental nutrition on resource-holding potential and resource valuation—and hence the direction of the relationship between developmental nutrition and aggression—remains unclear. Resource-rich developmental nutrition often increases adult body mass, and larger individuals are more likely to initiate and win aggressive contests in many species (Hoffman 1987; Shackleton et al. 2005; Briffa and Sneddon 2007; Brown et al. 2007; Bath et al. 2018). Likewise, high nutrient availability during development can increase relative resource allocation to traits (such as weapons) that enhance aggressive ability (Monaghan 2008; Colasurdo et al. 2009). Alternatively, resource-rich developmental nutrition might decrease aggressive motivation and the fitness benefits an individual gains from attaining a resource, through effects on the internal state (Arnott and Elwood 2008; Elias et al. 2010; Bath et al. 2018). For example, a lack of nutritional resources can increase motivation to attain food (Arnott and Elwood 2008). Likewise, if resource-poor nutritional conditions decrease lifespan (Good and Tatar 2001; Tigreros 2013), then they might increase motivation to attain access to breeding sites and mates (i.e. terminal investment; Clutton-Brock 1984; Krams et al. 2015; Moatt et al. 2016).

The existing studies that have investigated the relationship between developmental nutrition and aggression report contrasting responses across species. In the African striped mouse, *Rhabdomys dilectus chakae*, early-life protein deficiency leads to increased aggressive behaviour (Pillay et al. 2016), and low food availability increases aggressive lunging in the monarch caterpillar *Danaus plexippus* (Collie et al. 2020). However, in the southern field cricket *Gryllus bimaculatus*, a high-protein developmental nutrition increases aggression (Han and Dingemanse 2017), and aggression is higher in Argentine ant colonies (*Linepithema humile*) that develop on carbohydrate-rich diets (Grover et al. 2007). Furthermore, variation in developmental nutrition amongst individuals can influence aggressive interactions by generating asymmetries in fighting ability amongst rivals (Parker 1974; Briffa and Sneddon 2007; Asahina 2017), but few studies have evaluated the effects of developmental nutrition on both focal and rival individuals. Hence, the strength and direction of developmental nutrition effects on adult aggression remain largely unknown.

We used the fruit fly, *Drosophila melanogaster*, to investigate how the diet experienced during development influences adult male aggressive strategies. *Drosophila melanogaster* serves as an important model organism for both aggression and nutrition. Male *D. melanogaster* engage in frequent contests over mates and food patches, and success in contests influences mating success (Kravitz and Fernandez 2015). Access to food patches can provide increased nutrition, but, because males display only limited adult feeding (Carvalho et al. 2006), access to food patches largely provides access to mates (Dow and von Schilcher 1975; Hoffman 1987; Hoffmann 1987; Chen et al. 2002). Aggressive behaviours range from wing threat displays and fencing spars with forelegs to lunging, the principal aggressive behaviour, in which a male rears up and thrusts his upper body at his opponent (Hoffmann 1987; Chen et al. 2002; supplementary table 1). As a holometabolous insect with a juvenile food-acquiring stage distinct from the adult stage, the nutrition received during early life is critical to development in *D. melanogaster* (Boggs 1981). For example, early-life nutrition strongly impacts viability, body mass and post-copulatory reproductive traits (e.g. Gebhardt and Stearns 1993; Bross et al. 2005; McGraw et al. 2007). However, despite the wealth of knowledge on both nutrition and agonistic contests in this species, the direct link between developmental nutrition and adult aggressive behaviours has not been fully explored. Because *D. melanogaster* is a leading model organism for the neurobiology and physiology of aggression (Asahina 2017), understanding the relationship between developmental nutrition and aggression in this species would help to establish a system for in-depth investigation of the mechanisms linking nutrition and aggression.

We subjected male flies to low-, medium- or high-resource diets during larval development by manipulating yeast levels and then measured their adult aggressive behaviours. Yeast is an important source of protein, which is a key component of developmental nutrition for herbivorous insects that can be limiting under natural conditions (Lihoreau et al. 2015; Han and Dingemanse 2017). To test the effects of both an individual’s diet and the diet of its rival, we set up contests between pairs of males from all combinations of diet treatment in a fully factorial experimental design. We predict that if developmental diet influences aggression through effects on resource-holding potential, then high-resource developmental diets will increase adult aggression, whereas if developmental diet influences aggression through increased resource valuation, then low-resource developmental diets will increase aggression.

## Methods

### Experimental flies

All flies were derived from an outbred wild-type Dahomey stock population that has been maintained since 1970 in cages with overlapping generations (Carazo et al. 2015). Our use of an outbred population gives us the ability to explore variation in multiple aggressive traits. Stock populations were maintained on a standard molasses-based media (supplementary table 2). Fly husbandry was carried out at 25 °C on a 12:12 h light:dark cycle.

To generate experimental males, we collected eggs from the stock population using grape-agar plates smeared with yeast, transferred them at a standard density (50 eggs/vial) to vials containing food media (low, medium or high protein) and incubated them until eclosion. For ease of diet manipulation, we modified a simple sugar-yeast-agar medium (yeast:sugar ratio of 2:1) to create a ‘low’ (L; 10% yeast), ‘medium’ (M; 20% yeast) and ‘high’ resource media (H; 120% yeast; supplementary table 3). Yeast is the main source of protein in *D. melanogaster* diets and also contains micronutrients (e.g. vitamins, nucleic acids and cholesterol; Sang 1978). The optimal protein to carbohydrate ratio for male *D. melanogaster* larvae is between 1.5:1 and 2:1 (Rodrigues et al. 2015; Jang and Lee 2018), suggesting that our medium- and low-yeast media were substantially suboptimal in protein and that our high-yeast media contained slightly above optimal protein. We refer to these treatments as resource-poor or -rich to reflect that yeast manipulation alters protein, caloric and micronutrient content. Preliminary tests showed that these differences in yeast level were sufficient to generate differences in developmental duration and adult body mass. Developmental duration was extended as protein quantity was reduced, so egg collections for each treatment were staggered to synchronise adult eclosion.

We collected adult males using ice anaesthesia within 6 h of eclosion to ensure virginity, and transferred them to vials containing a standard molasses-based media, housing them individually to prevent the formation of social hierarchies that might influence aggressive behaviour (Penn et al. 2010; Trannoy et al. 2016). To differentiate males in behavioural trials, we painted each male with a small dot of red or white acrylic paint on the dorsal thorax between 1 and 4 days post-eclosion. Similar paint treatments had no detectable effect on behaviour in previous studies (Nilsen et al. 2004; Morimoto et al. 2016).

### Behavioural trials

Before trials, we deprived flies of food for 2 h in vials containing moist cotton wool to prevent desiccation. We then transferred pairs of males via gentle aspiration into observation chambers (20-mm diameter, 5-mm depth) containing a food patch of molasses-based media combined with yeast paste (5-mm diameter), a standard protocol for assessing aggression in *D. melanogaster* (Dierick 2007). In each pair, we arbitrarily designated one male as the focal male and the other as the rival male. We paired males in all combinations of treatments in a fully factorial design with 20–21 pairs per combination (supplementary table 4). To avoid confounding effects of paint colour, we painted half of the focal males red and the other half white. After a 5-min acclimatisation period, we recorded behaviour using a video camera (Toshiba Camileo X400) for 15 min. We conducted behavioural trials between 2 and 7 h Zeitgeber time over 4 days, with each individual fly trialled only once.

A single observer blind to treatment scored the videos using JWatcher (v. 1.0, Blumstein and Daniel 2007) and recorded the duration and number of occurrences of five aggressive behaviours (fencing, chasing, tussling, lunging and wing threat; supplementary table 1) for each focal and rival male. We also recorded the total locomotion duration and whether behaviours were performed on or off the food patch to allow testing for differences in locomotion and food patch access.

After trials, we froze and weighed males to assess the influence of nutrition on body mass. We weighed flies immediately after freezing to assess their mass during trials (wet mass), and weighed them again after they were dried for 48 h at 60 °C (dry mass).

### Statistical analyses

We performed analyses in R version 3.6.2 (2019–12-12). We expressed behavioural data as total durations (in seconds) or number of bouts of each behaviour. To test the influence of focal male diet, rival male diet and their interaction on focal male aggression, we first analysed lunging behaviour, the principal form of aggression displayed by male *D. melanogaster* (e.g. Hoyer et al. 2008; Asahina et al. 2014). Because lunging data were zero-inflated (101 of 187 focal males lunged), we analysed the influence of developmental diet on the likelihood of lunging in a binomial general linear model, and then on lunge number (within the subset of flies that did lunge) using a negative binomial general linear model.

Wing threat behaviour is a non-contact threat component of male aggressive behaviour. We analysed the influence of developmental diet on the number of focal male wing threats using a negative binomial distribution.

We then analysed the influence of developmental diet on total aggression duration, summed over lunging, fencing, tussling and wing threat, using a general linear model. Because our findings for total aggression differed from those for lunging, we secondarily explored total aggression further by conducting general linear models for the duration of fencing, and likelihood and duration of chasing independently. Chasing data were also zero-inflated (58 of 187 focal males chased), so we analysed the influence of diet on the likelihood of chasing in a binomial general linear model, and then on chase durations in the subset of flies that displayed chasing using a Gaussian general linear model. Because there were only two incidents of tussling, we did not analyse tussling separately. Because diet might affect locomotion, which might impact aggressive encounters, we assessed the influence of focal and rival diet on locomotion in a linear model.

To assess the interdependence of behaviour of the two individuals in a pair, we used a chi-square test of independence to assess whether the likelihood of a focal male lunging was related to the likelihood of his rival lunging. We then assessed the influence of rival lunge number on focal lunge number in the subset of males that lunged, and the influence of rival total aggression duration on focal total aggression duration amongst all males in linear models. To test whether this relationship was influenced by developmental diet, we performed chi-square tests of independence for each focal developmental diet treatment separately, and we ran an additional model including focal and rival diet and their interaction with rival aggression on focal total aggression.

In male *D. melanogaster*, food patches provide access to mates and males aggressively defend these sites (Markow 1988; Hoffman and Cacoyianni 1990; Lim et al. 2014). To test for differences in food patch occupancy, we analysed the influence of focal and rival developmental diet and their interactions on the amount of time focal individuals spent on the food patch using linear models. To evaluate how diet affects aggressive behaviour around food patches, we assessed the influence of focal and rival developmental diet on the proportion of aggressive behaviour that a focal individual performed on the food patch using general linear models fitted with the quasibinomial distribution.

We used diagnostic plots to assess model fit, and where data were over-dispersed, we used square root or log transformations to avoid violating the assumptions of parametric statistics. We included day as a fixed factor (Harrison et al. 2018) and Zeitgeber time as a covariate in all models to account for temporal variation in behaviour. We initially assessed models using type II ANOVA (analysis of variance) tests, and, when significant effects of developmental diet were detected, we explored the effect of diet further using post hoc Tukey tests. Because developmental diet might influence behaviour through effects on body mass, we first analysed the influence of developmental diet on wet and dry body mass using linear models. For aggressive behaviour that showed a significant response to focal or rival developmental diet, we further explored whether body mass had an effect on aggression above and beyond that of developmental diet, using simplified models including only focal or rival diet (depending on which had shown an effect in original models), and the corresponding mass, conducting sequential sum of squares analysis (type I ANOVA) to test the effect of mass after the main effects of developmental diet had been accounted for. We followed this by testing for effects of mass on these behaviours within each diet treatment group.

## Results

### Developmental diet influenced adult mass

**Fig. 1:**
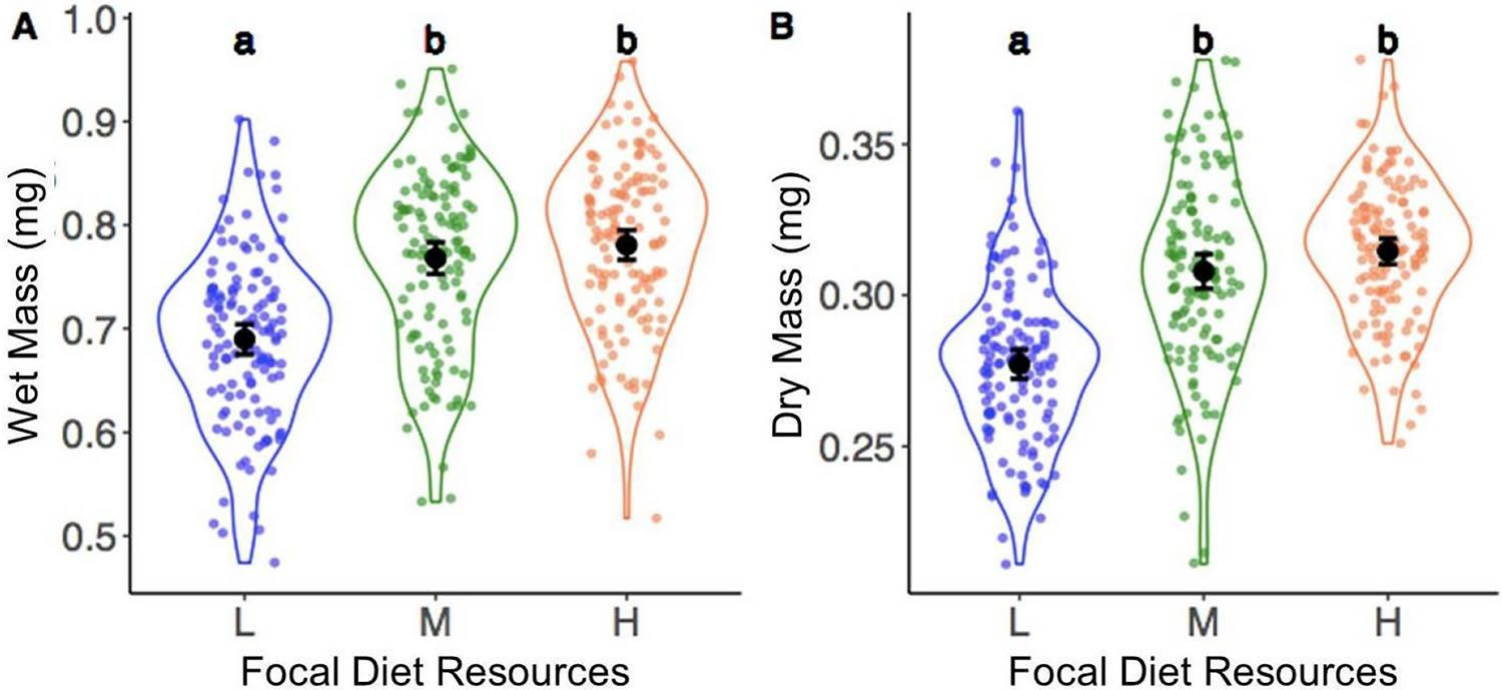
The wet mass (**A**) and dry mass (**B**) of adult males depending on their developmental diet. Black points show means; ‘violin’ areas represent the shape of the distribution; black bars show 95% confidence intervals

As expected, we found that developmental diet influenced adult mass (wet mass: F_2,373_ = 56.4, p < 0.0001; dry mass: F_2,370_ = 60.2, P < 0.0001; Fig. 1). Post hoc tests revealed that low-resource males were significantly lighter than medium and high-resource males (L-M comparisons: wet mass t =  − 8.3, p < 0.0001, dry mass = t =  − 8.2, p < 0.0001; L–H comparison: wet mass t =  − 9.9, p < 0.0001, dry mass = t =  − 10.4, p < 0.0001), which did not differ in mass (wet mass t = 1.7, p = 0.211; dry mass t =  − 2.2, p = 0.067), with a 12% reduction in both wet and dry mass between high- and low-resource males. These results demonstrate that males responded to our diet treatments.

### A resource-poor developmental diet reduced some aspects of aggression

**Fig. 2:**
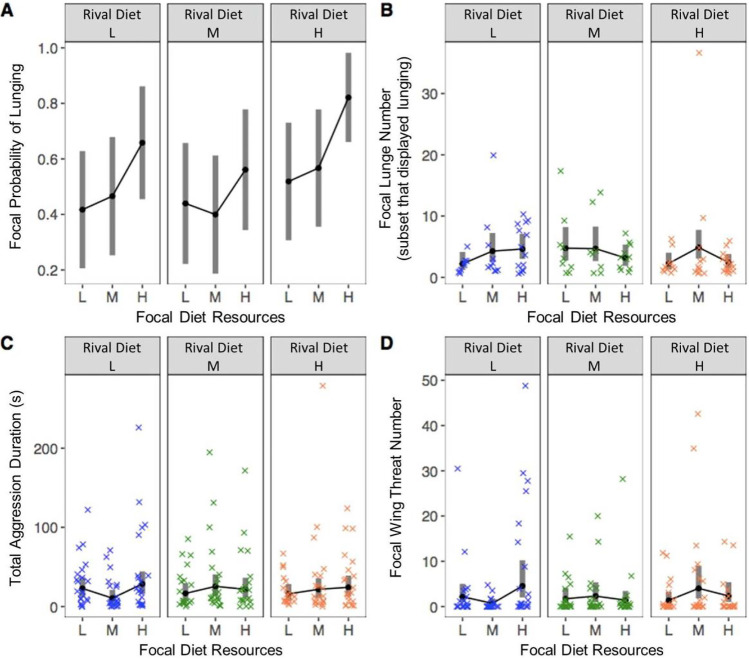
The influence of focal and rival developmental diet on focal male lunging probability (**A**), number of lunges (within males that displayed lunging, **B**), total aggression duration (in seconds, back-transformed from square root transformation, **C**) and wing threat number (**D**). Trials were 15 min long. Grey bars represent 95% confidence intervals

A focal male’s developmental diet influenced his likelihood of lunging (Table 1; Fig. 2a). Post hoc analyses revealed that high-resource males were more likely to lunge than low- and medium-resource males (M-H comparison: z =  − 2.4, p = 0.048; L–H comparison: z =  − 2.5, p = 0.030), but low and medium-resource males did not differ (z =  − 0.2, p = 0.976). However, in the subset of males that displayed lunging, focal male diet did not influence the number of lunges displayed (Table 1; Fig. 2b). We found no effect of a rival male’s developmental diet on focal male lunging, nor evidence of an interaction between a male’s developmental diet and that of his rival (Table 1; Fig. 2).

**Table 1:** The influence of focal and rival developmental diet and their interaction on focal aggressive behaviours

Behaviour	Focal diet	Rival diet	Focal diet x rival diet
Lunging probability	χ^2^_2,174_ = 8.1 **p = 0.018**	χ^2^_2,174_ = 4.1 p = 0.130	χ^2^_4,174_ = 1.1 p = 0.90
Lunge number (amongst flies that lunged)	χ^2^_2,88_ = 4.1 p = 0.132	χ^2^_2,88_ = 2.1 p = 0.335	χ^2^_4,88_ = 6.5 p = 0.162
Wing threat duration	χ^2^_2,174_ = 8.0 **p = 0.018**	χ^2^_2,174_ = 0.6 p = 0.759	χ^2^_4,174_ = 10.6 **p = 0.032**
Total aggression duration	F_2,174_ = 1.0 p = 0.364	F_2,174_ = 0.03 p = 0.968	F_4,174_ = 1.3 p = 0.273

Bold values indicate significance at α = 0.05

Our data do not support the hypothesis that high-resource males lunge more often solely as a consequence of their larger body mass, as focal male lunging probability was not related to focal male mass (χ^2^_1_ = 0.02, p = 0.886), and sequential sums of squares analysis revealed no further effect of focal body mass after focal male developmental diet was accounted for (wet mass F_1,179_ = 0.2, p = 0.653; dry mass F_1,179_ = 1.91, p = 0.168). Within the low-resource treatment, focal males of a lower dry mass were less likely to lunge (χ^2^_1,54_ = 6.4, p = 0.011), although there was no within-treatment effect of wet mass (χ^2^_1,54_ = 2.2, p = 0.138). Within medium and high-resource treatments, focal mass had no influence on lunge probability (medium-resource: dry mass χ^2^_1,55_ = 1.4, p = 0.243; wet mass χ^2^_1,55_ = 2.4, p = 0.118; high-resource dry mass χ^2^_1,54_ = 0.6, p = 0.443; wet mass χ^2^_1,54_ = 0.1, p = 0.814).

We found no detectable effect of the developmental diet of focal and rival males on total aggressive behaviour or on chasing and fencing (Table 1; supplementary Fig. 1). Because high-resource males lunged more often but did not display more total aggression, we wondered whether high-nutrition males performed other aggressive behaviours less frequently. However, we found no significant effect of developmental diet on other aggressive behaviours (Table 1; supplementary table 5; supplementary Fig. 1). Some behaviours varied with time and day (supplementary table 6), but treatments were dispersed across times and days and models included time and day to control for this variation.

Males of a high-resource developmental diet showed increased wing threat compared with males that developed on a lower-resource diet, but only when paired with rivals of a low-resource diet (Table 1; Fig. 2c). This pattern was not explained by differences in mass, as we found no effects of focal or rival male mass on wing threat number (focal male mass: χ^2^_1,176_ = 0.0, p = 0.892; rival male mass: χ^2^_1,176_ = 0.0, p = 0.862) and no interaction between focal and rival mass (χ^2^_1,176_ = 0.1 p = 0.803). Furthermore, sequential sums of squares analysis revealed no interaction between focal and rival body mass after focal and rival developmental diet were accounted for (interaction between focal and rival wet mass: F_1,170_ = 0.2, p = 0.648; interaction between focal and rival dry mass: F_1,168_ = 0.5, p = 0.472).

Threat displays can represent strategies to settle contests without costly escalation. However, we found no evidence that wing threat reduced escalated fighting, as there was a positive correlation between lunge number and wing threat number (Kendall’s rank correlation τ = 0.3, z = 5.5, p < 0.0001; supplementary Fig. 2).

The influence of developmental diet on lunging and wing threat could not be explained by differences in locomotion, as we detected no differences in locomotion duration related to developmental diet (focal diet F_2,174_ = 1.4, p = 0.240; rival diet F_2,174_ = 2.6, p = 0.079; supplementary Fig. 3).

### The developmental diet of rivals influenced aggression performed on the food patch

**Fig. 3:**
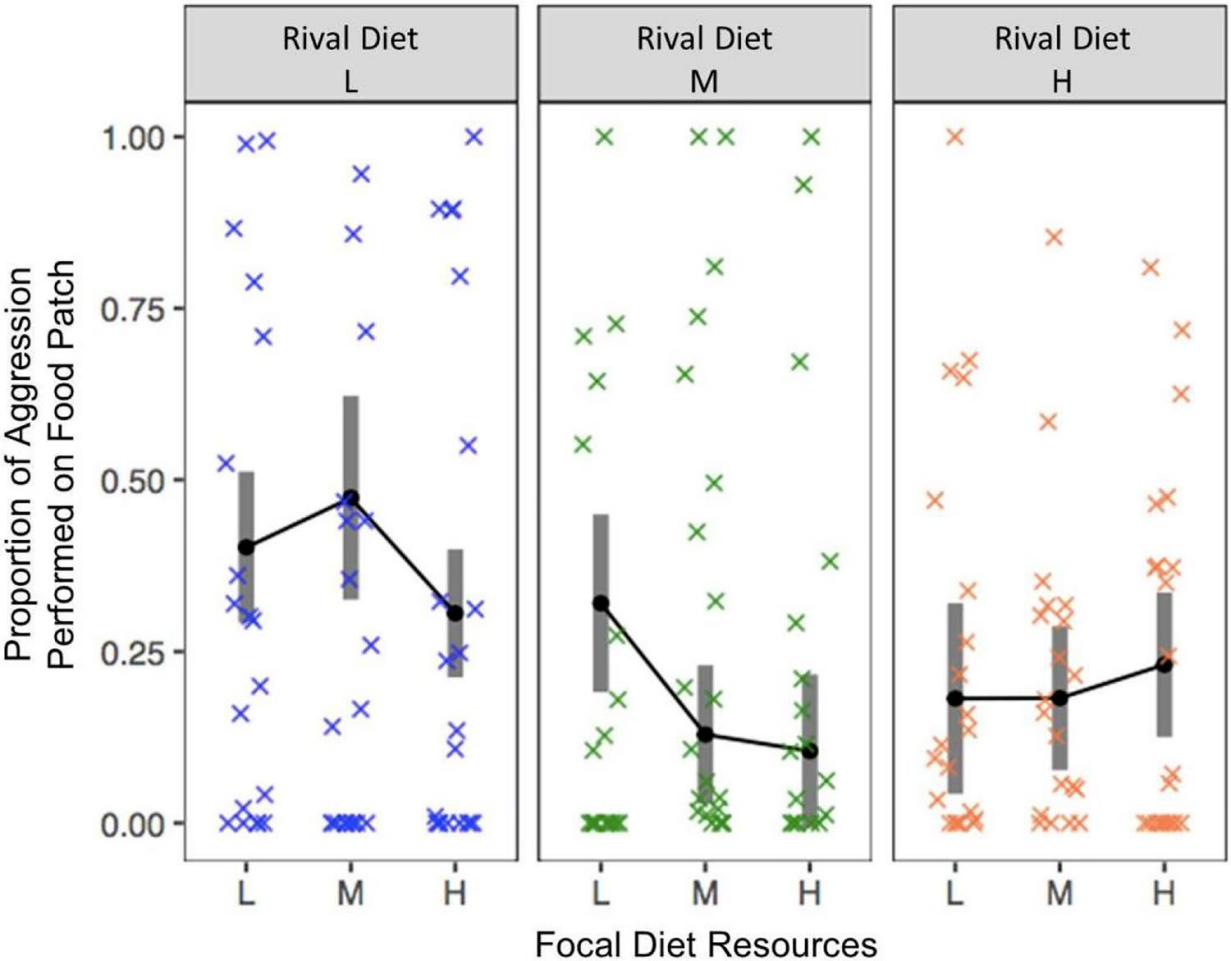
The influence of focal and rival developmental diet on the proportion of aggression the focal male performed on the food, relative to off the food. Grey bars represent 95% confidence intervals

We investigated how aggression related to access to the food patch because food patches represent valuable breeding sites for male *D. melanogaster.* We detected no effect of developmental diet on the time focal males spent on the food patch (focal diet: F_2,174_ = 0.6, p = 0.552, rival diet: F_2,174_ = 0.1, p = 0.867, interaction: F_4,174_ = 0.8, p = 0.519). However, focal males performed relatively more of their aggression on the food patch (as opposed to off the food patch) when competing against rivals of a low-resource developmental diet (χ^2^_2,168_ = 18.9, p < 0.0001; Fig. 3), but neither focal diet nor the interaction between focal and rival diet had a detectable effect (focal nutrition: χ^2^_2,168_ = 3.4, p = 0.182; interaction: χ^2^_4,168_ = 8.1, p = 0.089). Although focal males displayed relatively more of their aggression on the food patch as rival mass decreased (χ^2^_1,172_ = 5.3, P = 0.021; supplementary Fig. 4), sequential sums of squares analysis revealed no further effect of rival mass after rival developmental diet was accounted for (rival wet mass F_1,178_ = 0.8, p = 0.474; rival dry mass F_1,176_ = 0.2, p = 0.849).

### Aggression levels are not correlated within pairs

Because rival behaviour might influence focal male behaviour, we examined the relationship between the two. Chi-square analysis revealed that the probabilities of a focal and rival male performing at least one lunge are not independent (χ^2^_1_ = 18.5, p < 0.001). We found some evidence that diet influences this relationship, because although focal and rival male lunging was related in the low- and medium-resource focal developmental diet treatments (low: χ^2^_1_ = 7.7, p = 0.006; medium: χ^2^_1_ = 4.5, p = 0.034), we found no relationship the high-resource focal developmental diet treatment (χ^2^_1_ = 2.3, p = 0.126). Amongst those focal males that lunged, rival lunge number did not influence focal lunge number (χ^2^_1,95_ = 0.5, p = 0.470). Furthermore, the total duration of focal male aggression was not related to the total duration of rival aggression (F_1,181_ = 1.2, p = 0.277), and this was not influenced by differences in developmental diet (duration of rival aggression × focal diet interaction: F_2,165_ = 0.2, p = 0.855, duration of rival aggression × rival diet interaction: F_2,165_ = 0.3, p = 0.724).

## Discussion

We found that developmental diet influenced adult male aggression in *D. melanogaster*: a low-resource developmental diet reduced the likelihood of aggressive lunging (against all rivals) and wing threats (against rivals of low-resource diet). The influence of developmental diet on aggression appears above and beyond any influence of body mass. The developmental diet of rival males influenced where males performed aggression: focal males concentrated their aggression to the food patch when competing with low-resource rivals, suggesting that males may be better able to access food resources against nutritionally poorer rivals. Our findings suggest that adult *D. melanogaster* alter aggressive behaviour in light of their own developmental diet, through factors distinct from body mass, and that the nutritional experience of social partners also impacts contest characteristics.

The hypothesis that a resource-poor developmental diet would restrict growth and allocation to traits that mediate aggression (Monaghan 2008) predicts that a resource-poor developmental diet should decrease adult aggression. The reduced lunging probability of males subjected to low resource during development supports this prediction. However, although a low-resource developmental diet reduced adult body mass, in line with previous findings (Zikovitz and Agrawal 2013), differences in body mass did not explain the relationship with aggression, over and above the effect of developmental diet. This suggests that the relationship is mediated through mass-independent effects of diet, such as changes to internal state and energy budget. There was substantial mass variation amongst treatments, suggesting that insufficient variation did not explain the absence of mass effects beyond diet effects. Our results suggest that underlying variation in the developmental environment might explain the positive association between mass and aggression found in both male and female *D. melanogaster* (Hoffman 1987; Markow 1988; Hoyer et al. 2008; Bath et al. 2018) as well as other invertebrates (Shackleton et al. 2005; Brown et al. 2007) and vertebrates (reviewed by Briffa and Sneddon 2007).

Developmental diet might play a larger role in determining aggression than body mass per se does because diet can influence a range of physiological factors including resource allocation, energy levels and the relative growth of different traits. Indeed, in male *Drosophila*, developmental diet can have wide-ranging impacts, with resource-poor developmental diets reducing a male’s ability to transfer sperm and induce a refractory state in mates (McGraw et al. 2007), reducing his courtship success (Sharp and Agrawal 2009; Morimoto et al. 2016; Wigby et al. 2016), and reducing his success in post-copulatory sperm competition (Bangham et al. 2002; Morimoto et al. 2016). These effects can also be independent of the influence of diet on mass (McGraw et al. 2007). Furthermore, in other species, nutrition-induced correlates of condition, such as resting metabolic rate and energy reserves, can better predict aggression than body mass does (e.g. in the freshwater prawn *Macrobrachium rosenbergii*, Brown et al. 2003 swordtail *Xiphophorus helleri*, Royle et al. 2005, and damselfly *Calopterya splendens xanthostama*, Plaistow and Siva-Jothy 1996). If developmental nutrition does cause differences in physiology, then males that develop on resource-poor diets might adopt alternative strategies to maximise fitness returns from their limited energy reserves, rather than engaging in contests they are likely to lose.

We found that some aspects of male aggression—including chasing and fencing—did not show the same response to developmental diet as did lunging. The high intensity of lunging might make it more sensitive to developmental resource levels than less intense aggressive behaviours. Furthermore, the results suggest that developmental diet influences the propensity to engage in high-intensity lunging, but has less influence on the number of lunges required to resolve a contest. These results highlight how measuring a single aggressive behaviour might not capture the full picture of how ecological factors influence aggression (Chen et al. 2002; Alekseyenko et al. 2010; Certel and Kravitz 2012). Our finding that developmental diet has varying influences on different aspects of aggression is consistent with results for mating behaviour in *D. melanogaster*. The developmental environment influences several male sexual traits (as described above; Bangham et al. 2002; McGraw et al. 2007; Wigby et al. 2016), but other sexual traits such as sperm length (Amitin and Pitnick 2007) and mating rate, duration and latency (Lefranc and Bundgaard 2000; Edward and Chapman 2012) show little or no sensitivity. Thus, different aspects of multifaceted behaviours, such as aggressive and sexual behaviours, might be free to vary independently, allowing fine-tuned responses to ecological cues. Interestingly, nutritionally stressful developmental conditions can increase larval cannibalism—a potential form of developmental aggression—in *D. melanogaster* (Vijendravarma et al. 2013). When taken with our finding of reduced adult aggressive lunging after a resource-poor developmental diet, this suggests contrasting short-term and long-term effects of developmental nutrition on aggressive behaviours.

Nutrient quality and quantity can signal the nature of the prevailing social environment, providing information about mates, rivals and the costs and benefits of adult behavioural strategies (Enquist and Leimar 1987; Elias et al. 2010). Such information can influence behavioural motivation and resource valuation and prime individuals to cope with similar conditions as adults (Wigby et al. 2016). Our findings do not support the prediction that low-resource developmental diets increase aggression through increased resource valuation and motivation to compete aggressively for access to food patches (Elias et al. 2010; Bath et al. 2018). Alternatively, because moderate nutritional deprivation (i.e. that does not reach the severity of starvation) can increase lifespan in *D. melanogaster* (Pletcher et al. 2002; Klepsatel et al. 2018) and other species (Mair and Dillin 2008), flies that develop under resource-poor conditions might delay reproductive effort and have reduced motivation for early-life contests over breeding territories (Dow and von Schilcher 1975; Hoffman 1987; Hoffmann 1987; Chen et al. 2002). Future studies that explore the relationship between developmental diet, lifespan and aggression would be illuminating. The benefit of behavioural plasticity in response to environmental variation decreases as the duration between cue detection and the performance of the behavioural strategy increases (Fusco and Minelli 2010; Bretman et al. 2011a, 2011b). Hence, changes in resource valuation in response to adult nutrition might override any developmental nutritional experience (Edmunds et al. 2021). Further studies that consider the influence of adult nutrition on aggression would help resolve this question.

Surprisingly, males did not vary their level of direct physical fighting in response to the developmental diet of their rival. Contest theory suggests that physical fighting should be used sparingly against rivals of superior condition (Maynard Smith and Parker 1976; Bishop and Cannings 1978; Hammerstein and Parker 1982; Enquist and Leimar 1983; Leimar and Enquist 1984; Briffa and Sneddon 2007). Previous studies report that both male and female *D. melanogaster* regulate their aggression in response to opponent body mass (Hoffman 1987; Bath et al. 2018). However, the diet-induced variation in body mass in this study was relatively small and more representative of natural variation (e.g. a 12% difference in mass between low- and high-developmental diet males in these experiments, versus a 50% difference in Hoffmann, 1987). Because losing fights in *D. melanogaster* seldom results in direct physical damage to structures such as the wings (Guo and Dukas 2020), fighting behaviour might be less sensitive to small differences in rival conditions in this species. Our results support the view that fighting is instead primarily determined by an individual’s own developmental diet.

However, focal individuals did respond to the developmental diet of their rival in the relative amount of aggression performed on the food patch and in threat behaviour. Food patches can represent both nutritional sources and breeding sites (Markow 1988; Lim et al. 2014), so this result suggests that developmental diet and subsequent adult mass of a rival might influence a male’s ability to dominate access to these sites. Threat displays can allow individuals to assess or intimidate rivals without engaging in costly fights (Clutton-Brock and Albon 1979; Logue et al. 2010). Interestingly, recent work in *D. melanogaster* has demonstrated that populations evolved on a low-carbohydrate, high-protein diet in one laboratory favour wing threats over lunges in establishing dominance, whereas those evolved on a higher-carbohydrate, low-protein diet in another laboratory display more lunges, suggesting these two behaviours might represent alternate strategies (Legros et al. 2021). However, it is not obvious from our results that wing threats functioned as a strategy to avoid lunging. At face value, our findings that males that developed on a high-resource diet performed both more wing threats and more aggressive lunging do not suggest that the use of wing threats reduced lunging, but it is not possible to tell from our data if flies would have lunged more often had they not used these threat displays. Furthermore, it is possible that in natural settings, or in the large population cages in which these males recently evolved, threat displays by high-resource males might cause low-resource rivals to flee, avoiding escalated conflict, but this was not possible in our observation chambers.

Previous research has demonstrated that multiple aspects of the *D. melanogaster* diet can influence male fighting and reproductive behaviour throughout life. For example, excess saturated and trans-fatty acids in the adult diet can induce a more aggressive state (Meichtry et al. 2020), food deprivation increases male aggression (Edmunds et al. 2021), and developmental nutrition can influence post-copulatory male-male competition (McGraw et al. 2007). Our results add to this growing body of literature by providing evidence of an influence of developmental yeast levels on aggression. We demonstrate that, under our experimental conditions, an individual’s direct aggression is primarily determined by its own developmental diet, but the developmental experience of the rival influences threat behaviour and the defence of food patches during contests. Our findings contribute to the view that early-life experiences, particularly nutritional experience, shape behaviour throughout life (Monaghan 2008; Gluckman et al. 2016). Given the extensive molecular genetic approaches available in this model organism (Asahina 2017), our results offer the opportunity for future exploration of the mechanisms underlying nutritional regulation of aggression that could be applied on a broader taxonomic scale. In humans, the balance of nutrients received during childhood can influence aggressive behaviours later in life (Liu et al. 2004; Galler et al. 2012; Collie et al. 2020). Uncovering the ecological factors that determine aggression can help us understand variation in antagonistic behavioural strategies and predict social dynamics, and, in a human context, might help to limit the negative consequences of aggression.

## Supplementary Information

Below is the link to the electronic supplementary material.

Supplementary file1 (PDF 499 KB)

## Data Availability

Data are available as supplementary material.
