# Supplementary material for: A resource-poor developmental diet reduces adult aggression in male Drosophila melanogaster
Source: Behav Ecol Sociobiol. 2021 Jul 22;75(7):110. doi: 10.1007/s00265-021-03050-z (PMC8549984; doi:10.1007/s00265-021-03050-z)
Supplement: Supplementary file 1 — Supplementary file1 (PDF 499 KB) [file 265_2021_3050_MOESM1_ESM.pdf]

Supplementary Information

Supplementary Tables

Supplementary Table 1. A description of male aggressive behaviours (Nilsen et al, 2004, Dow & von Schilcher, 1974, Chen et al. 2002). Lunging, chasing, tussling and fencing all represent aggressive fighting, whereas wing threat represents an aggressive threat display.

**Aggressive behaviours**	**Description**
Lunging	*The male raises up on hind legs and rapidly thrusts his upper body at his opponent*
Chasing	*One male rapidly pursues his opponent, remaining in close proximity. Contact and even aggressive lunging may occur during chasing*
Tussling	*Both opponents raise up on hind legs and become interlocked in a prolonged aggressive struggle*
Fencing	*A male uses his forelimbs to bat his opponent. It includes front-on and side-on action with any of the two front or middle legs, and can be performed by one or both opponents, alone or in combination with other behaviours.*
Wing Threat	One male faces the rival and raises both wings in unison at an angle of ~45 degrees for a duration of >1s

Supplementary Table 2. Standard molasses-based medium laboratory medium for adult flies

**Ingredients**	**Amount Per Litre**
Agar (g)	6.923
Maize (g)	69.231
Soya (g)	8.308
Yeast (g)	14.077
Malt (g)	69.231
Molasses (ml)	23.077
Nipagin (ml)	30.000
Acid mix (propionic acid + orthophosphoric acid, ml)	5.385

Supplementary Table 3. Larval food recipes adjusted from a sugar-yeast-agar recipe (values per litre media)

**Ingredients**	**Amount Per Litre**		
	**High Yeast**	**Medium Yeast**	**Low Yeast**
Water (ml)	960	960	960
Agar (g)	15	15	15
Sugar (g)	50	50	50
Yeast (g)	120	20	10
Nipagin (ml)	30	30	30
Propionic acid (ml)	3	3	3

Supplementary Table 4. Sample sizes for each treatment

Focal Diet	Rival Diet	N
L	L	21
L	M	20
L	H	21
M	L	21
M	M	21
M	H	21
H	L	21
H	M	20
H	H	21

Supplementary Table 5. The influence of focal and rival developmental diet and their interaction on focal male fencing and chasing aggressive behaviours

Behaviour	Focal diet	Rival diet	Focal diet x rival diet
Fencing duration	$F_{2,174}$=1.2 p=0.291	$F_{2,174}$=0.1 p=0.954,	$F_{4,174}$=0.8 p=0.504
Chasing probability	$\chi^2_{2,174}$=0.3 p=0.879	$\chi^2_{2,174}$=1.1 p=0.592	$\chi^2_{4,174}$=2.5 p=0.645
Chase duration (among flies that chased)	$F_{2,45}$=0.9 p=0.431	$F_{2,45}$=0.1 p=0.892	$F_{4,45}$=0.7 p=0.619

Supplementary Table 6. The effects of Zeitgeber time and day from models of the influence of larval diet on each behaviour. We included day as a fixed factor and Zeitgeber time as a covariate in all models to account for temporal variation in behaviour.

Behaviour	Zeitgeber time	Day
**Lunge probability**	$\chi^2_{1,174}$=2.9, p=0.089	$\chi^2_{3,174}$=3.6, p=0.309
**Lunge frequency**	**$\chi^2_{1,88}$=4.8, p=0.029**	$\chi^2_{3,88}$=1.5, p=0.6378
**Wing threat**	$\chi^2_{1,174}$=1.9, p=0.662	**$\chi^2_{3,174}$=8.0, p=0.047**
**Locomotion**	$F_{1,174}$=3.2, p=0.077	**$F_{3,174}$=6.1, p<0.001**
**Fencing**	$\chi^2_{1,174}$=1.2, p=0.270	**$\chi^2_{3,174}$=3.0, p=0.031**
**Chase probability**	$\chi^2_{3,174}$=2.3, p=0.508	$\chi^2_{1,174}$=1.4, p=0.230
**Chase frequency**	$\chi^2_{3,45}$=0.5, p=0.707	$\chi^2_{1,45}$=0.0, p=0.995
**Total aggression**	$F_{1,174}$=1.9, p=0.167	$F_{3,174}$=2.2, p=0.091

Supplementary Figures

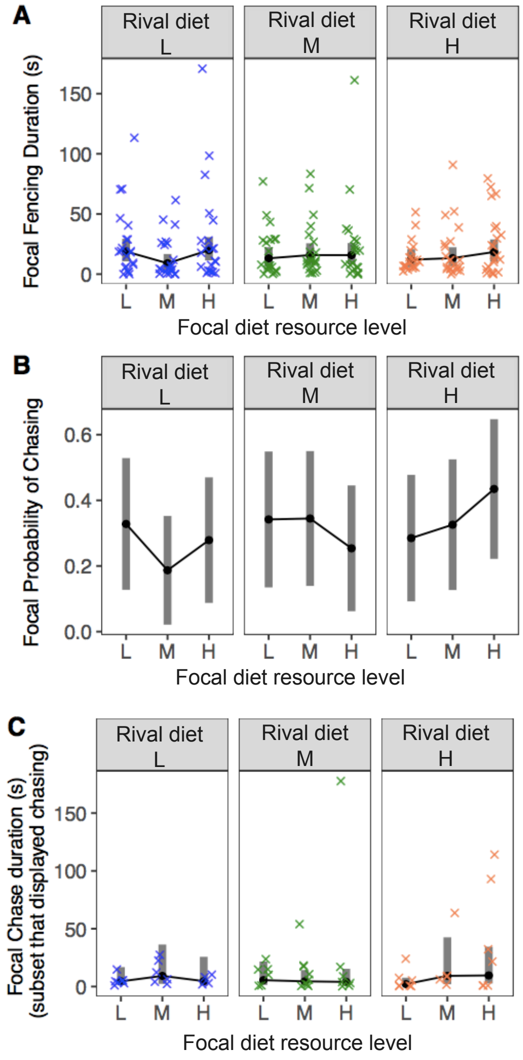

Supplementary Figure 1

The influence of focal and rival developmental diet on focal male fencing duration (back-transformed from square root transformation, A), chase probability (B), chase duration of those that chased (back-transformed from log transformation, C), Grey bars represent 95% confidence intervals.

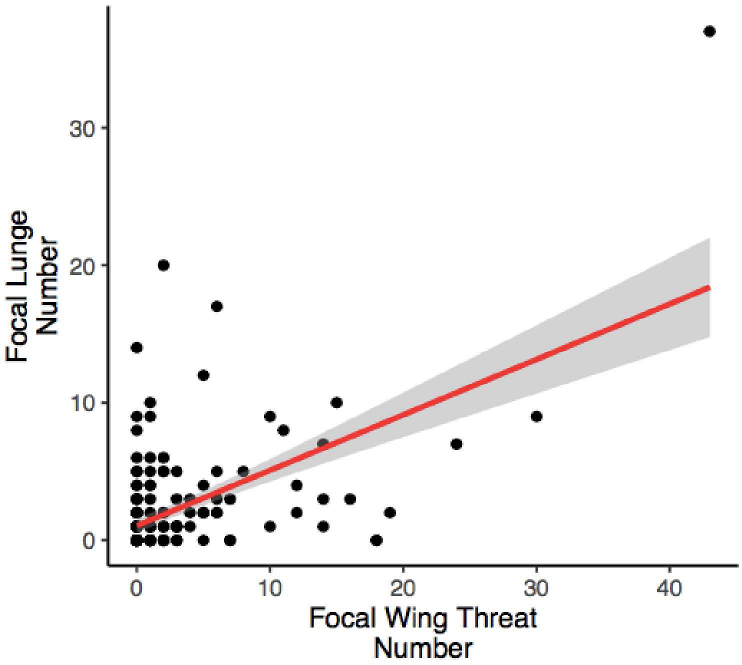

Supplementary Figure 2
The relationship between focal male wing threat number and lunge number. Black points show raw data; red line shows the line of best fit; grey shaded area represents confidence intervals

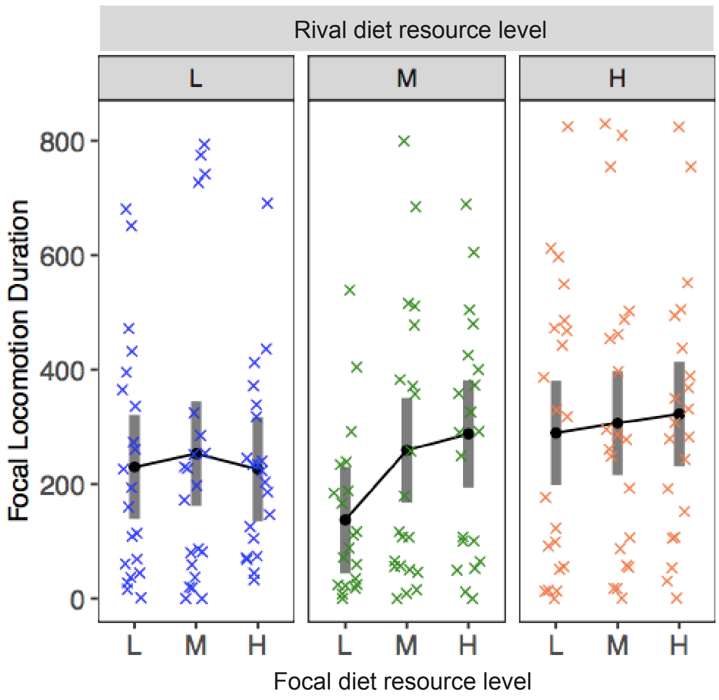

Supplementary Figure 3
The influence of focal and rival developmental diet on focal male locomotion duration. Grey bars represent 95% confidence intervals.

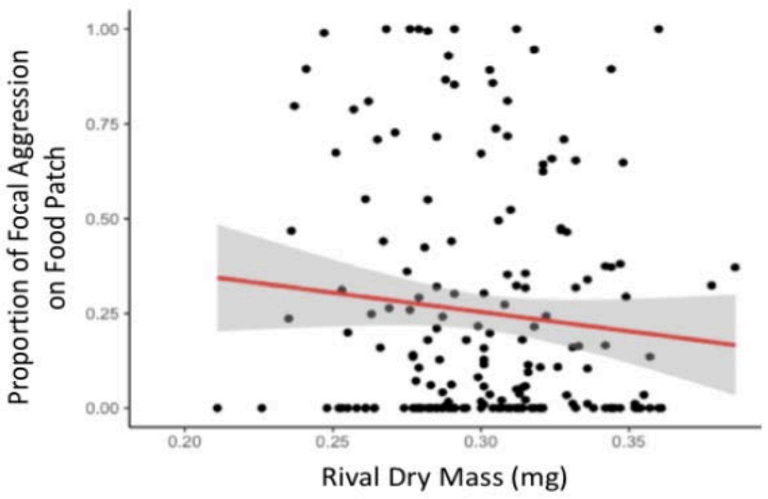

Supplementary Figure 4
The relationship between rival male dry mass and the proportion of aggression the focal male performed on the food patch, relative to off the food patch
